# Distribution Patterns for Bioactive Constituents in Pericarp, Stalk and Seed of Forsythiae Fructus

**DOI:** 10.3390/molecules25020340

**Published:** 2020-01-14

**Authors:** Lifang Wei, Yuqi Mei, Lisi Zou, Jiali Chen, Mengxia Tan, Chengcheng Wang, Zhichen Cai, Liqun Lin, Chuan Chai, Shengxin Yin, Xunhong Liu

**Affiliations:** College of Pharmacy, Nanjing University of Chinese Medicine, Nanjing 210023, China; weilifangquiet@163.com (L.W.); 18260028173@163.com (Y.M.); zlstcm@126.com (L.Z.); 18994986833@163.com (J.C.); 18816250751@163.com (M.T.); ccw199192@163.com (C.W.); caizhichen2008@126.com (Z.C.); quinn6182@163.com (L.L.); echo_0523@hotmail.com (C.C.); yinshengxin723@163.com (S.Y.)

**Keywords:** Forsythiae Fructus, pericarp, stalk, seed, bioactive constituents, distribution patterns

## Abstract

Forsythiae Fructus (FF) is a widely used folk medicine in China, Japan, and Korea. The distribution of bioactive constituents throughout the fruit segments has rarely been addressed, although mounting evidence suggests that plant secondary metabolites are synthesized and distributed regularly. The phytochemical profiles of three segments of FF (pericarp, stalk and seed) were firstly revealed by liquid chromatography-tandem mass spectrometry (LC-MS/MS)-based quantitative analysis of twenty-one bioactive constituents, including three phenylethanoid glycosides, five lignans, eight flavonoids, and five phenolic acids to explore the spatial distribution of bioactive constituents. Furthermore, the hierarchical clustering analysis (HCA) and one-way analysis of variance (one-way ANOVA) were conducted to visualize and verify the distribution regularity of twenty-one analytes among three segments. The results showed that phytochemical profiles of the three segments were similar, i.e., phenylethanoid glycosides covering the most part were the predominant compounds, followed by lignans, flavonoids and phenolic acids. Nevertheless, the abundance of twenty-one bioactive constituents among three segments was different. Specifically, phenylethanoid glycosides were highly expressed in the seed; lignans were primarily enriched in the stalk; flavonoids were largely concentrated in the pericarp, while the contents of phenolic acids showed no much difference among various segments. The research improves our understanding of distribution patterns for bioactive constituents in FF, and also complements some scientific data for further exploring the quality formation mechanism of FF.

## 1. Introduction

Forsythiae Fructus (FF), which is the dried fruit of *Forsythia suspense* (Thunb.) Vahl, has been utilized as a common traditional medicine in China, Japan, and Korea for the treatment of pyrexia, inflammation, gonorrhea, carbuncle, and erysipelas [1,2]. FF is also an effective heat-clearing and detoxifying medicine; indeed, 114 Chinese medicinal preparations containing FF are listed in the Chinese pharmacopoeia [3]. Hitherto, approximately 210 compounds have been reported from FF, including phenylethanoid glycosides, lignans, flavonoids, phenolic acids, terpenoids, cyclohexylethanol derivatives, and so on [4]. Among them, the first four types of compounds were demonstrated to be responsible for the diverse biological activities of the herbal medicine, such as antibacterial [5,6,7], anti-inflammatory [8,9], antiviral [10,11], antioxidant [12,13], anti-endotoxin [14], and hepatoprotective [15,16].

Previous studies have intensively described the effects of ecological environment [17,18], harvesting time [19], and processing methods [20] on the formation and accumulation of bioactive metabolites in FF, in virtue of chromatographic fingerprint techniques or absolute quantitative methods. Nonetheless, the location and relative abundance of bioactive constituents in various segments of the fruit, i.e., pericarp, stalk, and seed, remain unclear. There is growing evidence that, in the growth of medicinal plants, the synthesis and distribution of secondary metabolites have certain regularity [21,22,23]. Hence, it is rational to promote research to fill knowledge gaps regarding the metabolic diversity on the fruit segments of FF, so as to acquire a comprehensive understanding of the function of specific segments of the fruit.

The present study aims to elucidate the spatial distribution of bioactive constituents in FF. The contents of twenty-one major bioactive constituents in three segments of FF, including three phenylethanoid glycosides, five lignans, eight flavonoids, and five phenolic acids, were simultaneously determined by ultra-fast liquid chromatography coupled with triple quadrupole/linear ion trap tandem mass spectrometry (UFLC-QTRAP-MS/MS). Furthermore, the hierarchical clustering analysis (HCA) was introduced to observe the sample classification according to the contents of twenty-one analytes. One-way analysis of variance (one-way ANOVA) was performed to verify the abundance differences among three segments. This research provides helpful information regarding the distribution patterns of bioactive constituents in FF, which also complements some scientific data for further exploring the quality formation mechanism of FF.

## 2. Results

### 2.1. Optimization for Sample Extraction

Extraction variables, including extraction solvent ratio (30% methanol, 50% methanol, 70% methanol, 90% methanol, 100% methanol), liquid to material ratio (20:1 mL/g, 25:1 mL/g, 30:1 mL/g, 35:1 mL/g, 40:1 mL/g), and extraction time (15 min, 30 min, 45 min, 60 min) were firstly investigated by single factor experiment to obtain higher extraction efficiency (Figure S1). The sum of the extraction yields of forsythiaside A and phillyrin was taken as the response value, since these two compounds could be well separated and detected in all of the samples based on liquid chromatography. Subsequently, response surface methodology (RSM) based on the Box–Behnken design (BBD) was employed to optimize the extraction conditions (Table S1). The experimental results were fitted to a second-order polynomial equation and one-way ANOVA was used to estimate the goodness of fit of the model and determine the optimal conditions for responses (Table S2). According to Figure S2, the optimum extraction conditions were deduced, as follows: extraction solvent ratio, 72% methanol; liquid to material ratio, 38:1 mL/g; extraction time, 60 min. Triplicate confirmatory experiments were carried out under the optimum conditions to verify the reliability of the model. The total average extraction yield of forsythiaside A and phillyrin was 11.41 ± 0.01% with relative error from the theoretical value of 1.04%. The results showed that the optimized extraction conditions were reliable, so it was further applied for the extraction of FF.

### 2.2. Optimization of UFLC Conditions

For desirable resolution, key factors affecting chromatographic separation were fully optimized. Two types of chromatographic columns, Synergi^TM^ Hydro-RP 100 Å column (2.0 mm × 100 mm, 2.5 μm) and XBridge^®^C_18_ column (4.6 mm × 100 mm, 3.5 μm) were firstly examined by assessing the selectivity and column efficiency. It was found that the separation of target compounds was not much different by the two columns, while the chromatographic response of the latter was comparatively higher and its peak shape of the chromatogram was more symmetrical. Hence, the XBridge^®^C_18_ column was finally selected. Besides, three kinds of mobile phase systems, which consisted of water-methanol, water-acetonitrile, water (containing 0.1% formic acid)-acetonitrile on the chromatographic behavior of 21 analytes, were investigated. Consequently, water (containing 0.1% formic acid)-acetonitrile was chosen as the mobile phase with better separation and peak shape.

### 2.3. Optimization of MS Conditions

Individual solutions of all standard compounds were injected into the ESI source in both positive and negative ion modes in order to select proper instrumental parameters for MS/MS detection. The results showed that astragalin, phillyrin, and arctiin had higher sensitivity in positive ion mode, whereas other compounds exhibited a stronger response in negative ion mode. Thus, the ESI^+^ and ESI^-^ mode were simultaneously adopted in this experiment. Table 1 lists the optimized mass spectrometry parameters, including multiple-reaction monitoring (MRM) transitions, as well as declustering potential (DP) and collision energy (CE) of 21 analytes. Figure S3 provides typical MRM chromatograms of them.

### 2.4. Method Validation

The proposed UFLC-QTRAP-MS/MS method was validated by determining the linearity, limits of detection and quantification (LOD and LOQ), precision, repeatability, stability, and recovery. All of the analytes showed satisfactory linearities within corresponding concentration ranges with their correlation coefficients (*r*) all above 0.9990. The LODs and LOQs for all compounds were 0.001–233.710 ng/mL and 0.002–771.250 ng/mL, respectively, which indicated the high sensitivity of the method. The relative standard deviation (RSD) of precision, repeatability, and stability of all compounds ranged from 1.2% to 4.0%, 1.4% to 4.0% and 1.8% to 4.0%, respectively. The mean recoveries varied from 98.6% to 102% with RSDs less than 3.8%. The slope ratio values of standard addition calibration curves to solvent calibration curves were between 0.91 and 1.06, which indicated that the matrix effect on the ionization of analytes was not obvious under optimized conditions. The results demonstrated that the established method was qualified for quantitative analysis of target compounds. Table 2 presents detailed results.

### 2.5. Quantification of Bioactive Constituents in Various Segments

**Figure 1:**
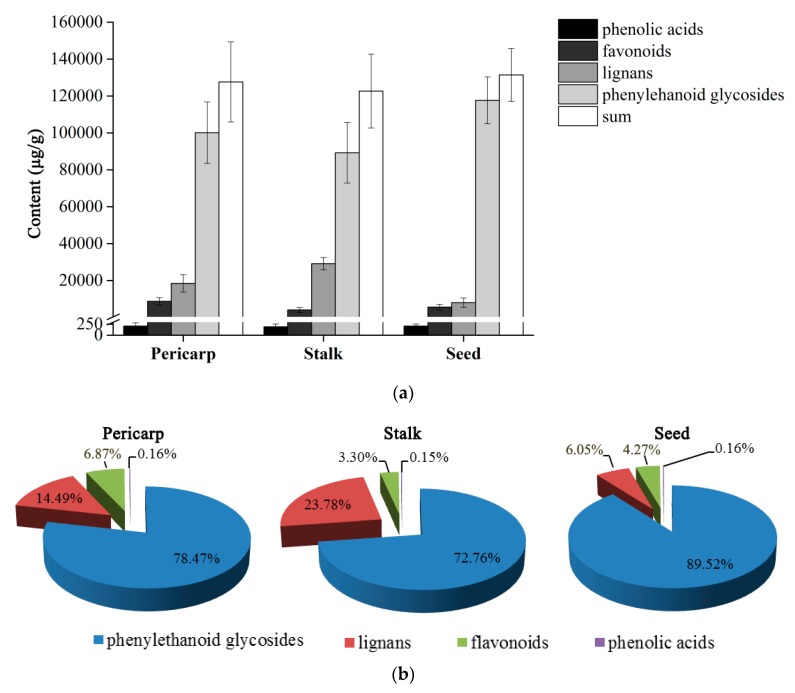
Content (**a**) and content ratio (**b**) of four structural types of analytes in different segments of Forsythiae Fructus.

The established UFLC-QTRAP-MS/MS method was subsequently applied to the simultaneous determination of bioactive constituents in different segments of FF. Contents of the twenty-one analytes were summarized in Tables S3–S5. As shown in Figure 1, four types of compounds analyzed could be detected in all segments of the fruit and they showed high diversity in content in the same segment. To be specific, the contents of phenylethanoid glycosides, which accounted for 72.76% to 89.52% of the total content of investigated compounds, were the highest, followed by lignans and flavonoids, and the contents of phenolic acids stayed at a low level. Moreover, it could be found that the phytochemical profiles among different segments were similar.

### 2.6. Distribution of Bioactive Constituents among Various Segments

**Figure 2:**
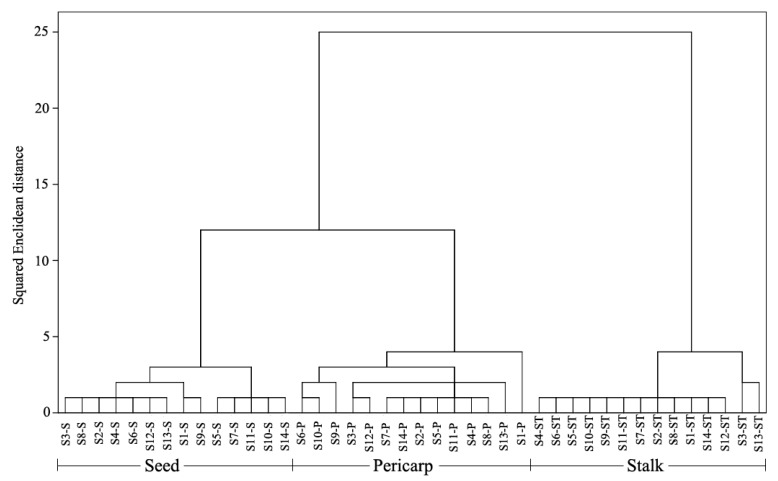
Dendrogram of three different segments from Forsythiae Fructus based on the content of twenty-one analytes (P, pericarp; ST, stalk; S, seed).

Additionally, the hierarchical cluster analysis, using the Ward’s method with Squared Euclidean distance as the similarity measurement was modeled to cluster the different groups according to the quantification of compounds analyzed. Figure 2 presents the resulting dendrogram. It could be found that samples from the same segment could be grouped into one cluster. Based on this, the “seed” samples and “pericarp” samples were initially clustered into one group and then clustered with “stalk” samples. When the dendrogram was cut with a horizontal line at the distance of 5, the samples could be classified into three categories, i.e., “seed” samples in a category, “pericarp” samples in a second category, and “stalk” samples in a third category.

**Figure 3:**
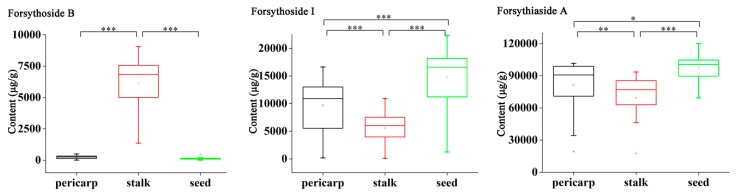

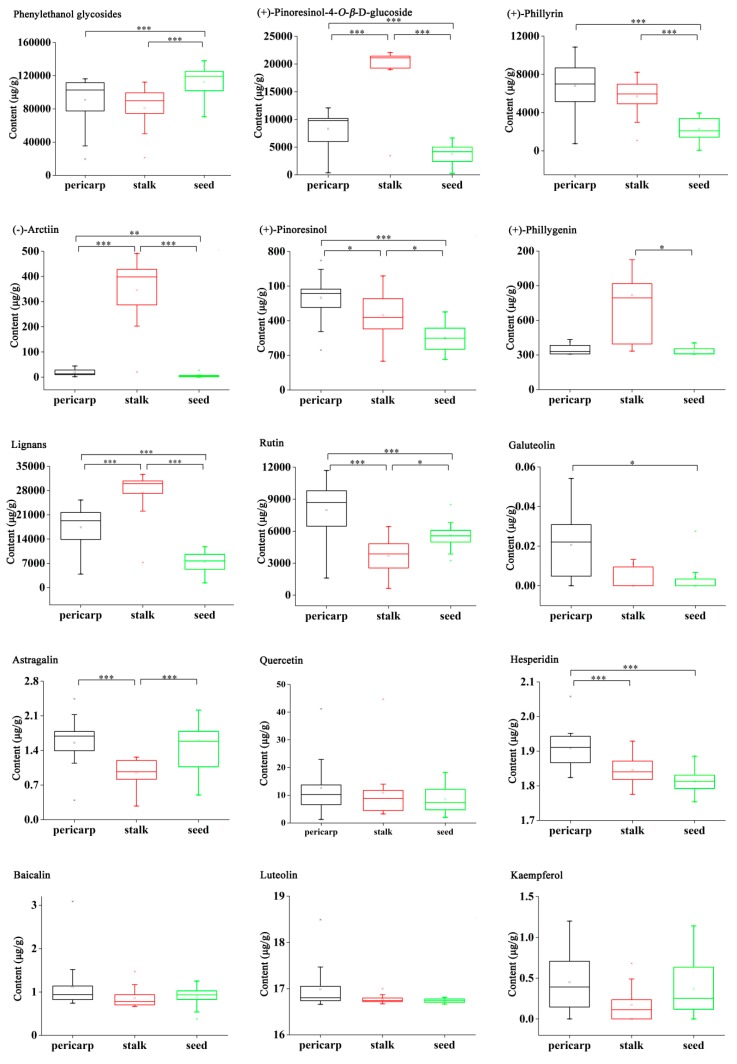

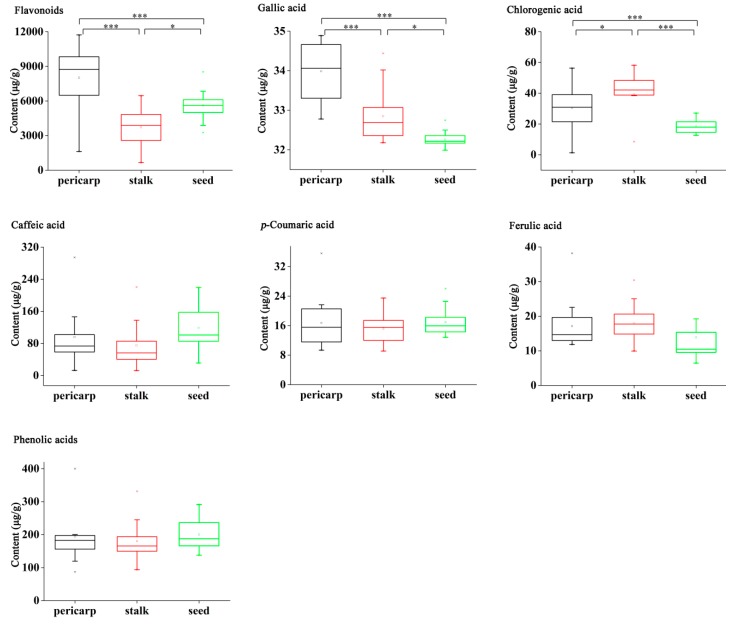
Variance analysis of twenty-one analytes among different segments of Forsythiae Fructus (* *p* < 0.05; ** *p* < 0.01; *** *p* < 0.005).

Furthermore, the one-way ANOVA followed by least significant difference (LSD) test (equal variance assumed) or Tamhane’s test (equal variance not assumed) was carried out to illustrate the abundance variation of twenty-one analytes among three segments. As shown in Figure 3, four-sevenths of compounds investigated demonstrated a significant difference among three segments of FF (*p* < 0.005). Notably, seed contained a dramatically higher amount of one phenylethanoid glycoside compound, forsyghoside I, than the other two segments of the fruit (*p* < 0.005). Stalk displayed a remarkably higher content of one phenylethanoid glycoside compound and two lignan compounds, that is, forsythoside B and pinoresinol-4-*O*-*β*-D-glucoside as well as arctiin (*p* < 0.005). Besides, the pericarp showed a significantly higher content of two flavonoid compounds and one phenolic acid compound, namely rutin, as well as hesperidin and gallic acid (*p* < 0.005). In terms of the total content of each structural type of constituents, a certain distribution tendency was also observed. Specifically, the seed contained an obviously higher amount of phenylethanoid glycosides (*p* < 0.005), stalk had a higher content of lignans (*p* < 0.005), while pericarp was found to be the richest part in flavonoids (*p* < 0.005). In addition, the total contents of phenolic acids showed not much difference among various segments.

## 3. Discussion

### 3.1. Selection of Analytes

Based on the comprehensive analysis of previous studies [24,25,26], twenty-five bioactive constituents, including phenylethanoid glycosides of forsythoside B, forsythoside I, and forsythiaside A; lignans of pinoresinol-4-*O*-*β*-D-glucoside, phillyrin, arctiin, pinoresinol, and phillygenin; flavonoids of rutin, hyperin, isoquercitrin, galuteolin, astragalin, quercetin, hesperidin, baicalin, luteolin, and kaempferol; as well as phenolic acids of gallic acid, chlorogenic acid, caffeic acid, *p*-hydroxybenzyl alcohol, *p*-coumaric acid, protocatechualdehyde, and ferulic acid were selected for the preliminary test. However, hyperin and isoquercitrin, a pair of isomers with very similar polarity and ion fragments, were finally eliminated, since they could not reach the baseline separation either by optimizing gradient elution conditions or transforming mass spectrometry detection channel. Besides, *p*-hydroxybenzyl alcohol and protocatechualdehyde, which were found to be of poor MS response and low concentration in samples, were also excluded. Finally, twenty-one compounds were selected as target analytes for UFLC-QTRAP-MS/MS analysis. The selected analytes basically cover all chemical structural types of bioactive constituents in FF [4], so their content variation can profile the distribution pattern of bioactive constituents in the fruit to a great extent.

### 3.2. Selection of Determination Method

Among the twenty-one target analytes, not only the compounds with extremely similar polarities exist, such as the isomers of phillyrin and arctiin, but also the contents of compounds display wide diversity, varying from 10% (forsythiaside A) to sub-ppm (galuteolin). The HPLC method is difficult to effectively separate compounds with similar polarities, and it has the disadvantages of time consuming and low sensitivity, so it could hardly quantify the above target analytes simultaneously. Triple quadrupole/linear ion trap mass spectrometry (QTRAP-MS/MS) combines multiple scanning functions of triple quadrupole mass spectrometry with multi-stage mass spectrometry capacity of ion trap mass spectrometry. It allows for substances with similar chromatographic behaviors to be completely separated under the multiple reaction monitoring mode in virtue of their differences in molecular weight or fragment mass. It also enables the simultaneous determination of multiple compounds, which greatly shortens analysis time [27]. In summary, this technique possesses remarkable superiority in selectivity, sensitivity, and analysis capability, so it has been widely used for the separation and analysis of metabolites in medicinal materials [28,29,30]. Herein, the UFLC-QTRAP-MS/MS technology was adopted for the simultaneous determination of twenty-one analytes in FF.

### 3.3. Quantitative and Distributive Analysis of Bioactive Constituents in Various Segments

It could be assumed that the phytochemical profiles of three segments showed certain similarity, according to the quantification of twenty-one analytes. Phenylethanoid glycosides covering the most part were their predominant compounds. Lignans and flavonoids took the second and third place, respectively. Whereas, the contents of phenolic acids in them were relatively low. The aforementioned assumptions were consistent with previous reports in the literature [26].

Hierarchical cluster analysis, which is one of the most commonly used multiple factor analysis methods, not only offers a visual representation of complex data, but also provides a method for assessing the similarity or dissimilarity among samples [31]. Although a number of different clustering methods are widely used, the approach and underlying assumptions of many of these methods are quite different, thus they might lead to different clustering results [32]. Seven clustering methods, including between-groups distance, within-groups linkage, nearest neighbor, furthest neighbor, centroid clustering, median clustering, and Ward’s method, were compared to achieve better clustering quality. The results showed that the application of Ward’s method during the cluster analysis contributed to better clustering results. Hence, the Ward’s method with Squared Euclidean distance was employed to establish the clusters. Consequently, the method exhibited better performance with a clear differentiation among “pericarp”, “stalk”, and “seed” samples. It could be deduced from the clustering results that there are certain differences in bioactive constituents among different segments of FF.

One-way ANOVA was further performed on the mean contents of twenty-one analytes in order to verify the abundance difference of various fruit segments. The significance threshold was lowered to *p* < 0.005 and 0.005 < *p* < 0.05 was defined as “suggestive evidence” to reduce the false positive rate [33]. The results indicated significant difference among the “pericarp”, “stalk” and “seed” samples with four-sevenths of compounds displaying significant differences (*p* < 0.005). Additionally, the distribution characteristics of single-component were similar to that of the total same type component, except for forsythoside B and gallic acid, which were with particular abundance in stalk and pericarp, respectively. Generally, phenylethanoid glycosides were highly expressed in the seed; lignans were primarily enriched in the stalk, flavonoids were largely concentrated in the pericarp, whilst the contents of phenolic acids showed not much difference among various segments.

## 4. Materials and Methods

### 4.1. Plant Materials

**Figure 4:**
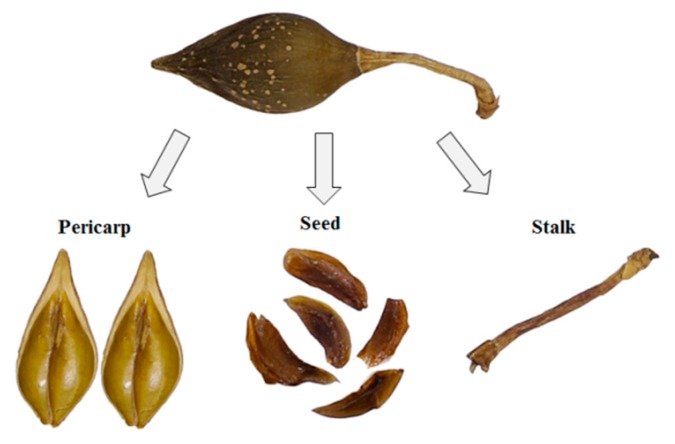
Three segments (pericarp, stalk and seed) of Forsythiae Fructus.

The plant materials were collected from the major producing areas of FF, including Shanxi, Shaanxi, and Henan provinces. Table 3 showed the detailed geographical origins for each sample. All of them were authenticated as Forsythiae Fructus (Qingqiao) by Prof. Xunhong Liu, School of Pharmacy, Nanjing University of Chinese Medicine, PR China. The voucher specimens were deposited at the identification laboratory of Nanjing University of Chinese Medicine. Subsequently, each of them was divided into three segments: pericarp, stalk, and seed (Figure 4).

### 4.2. Chemicals and Reagents

The reference standards of gallic acid (**1**), rutin (**6**), astragalin (**12**), and baicalin (**15**) were purchased from Chinese National Institute of Control of Pharmaceutical and Biological Products (Beijing, China). Chlorogenic acid (**2**) was received from Baoji Herbest Bio-Tech Co., Ltd. (Baoji, China). Caffeic acid (**3**), galuteolin (**9**), quercetin (**13**), and kaempferol (**19**) were provided by National Institutes for Food and Drug Control (Beijing, China). Forsythoside B (**4**), forsythoside I (**5**), forsythiaside A (**8**), (+)-phillyrin (**16**), and (+)-pinoresinol (**20**) were bought from Liangwei Bio-technology Co., Ltd. (Nanjing, China). *p*-Poumaric acid (**7**), ferulic acid (**10**), hesperidin (**14**), (−)-arctiin (**17**), luteolin (**18**), and (+)-phillygenin (**21**) were offered by Yuanye Bio-technology Co., Ltd. (Shanghai, China). (+)-Pinoresinol-4-*O*-*β*-D-glucoside (**11**) were supplied by Chengdu Chroma-Biotechnology Co., Ltd. (Chengdu, China). The purity of all standards was above 98% through liquid chromatography analysis. Figure S4 shows the chemical structures of the above twenty-one analytes. Merck offered Acetonitrile, Methanol and formic acid of chromatographic grade (Darmstadt, Germany). The deionized water was prepared by a Milli-Q water purification system (Millipore, Bedford, MA, USA).

### 4.3. Preparation of Standard Solutions

Each reference compound was accurately weighed and completely dissolved in 72% *(v/v)* methanol to produce their respective stock solutions, and their concentration was as follows: **1**, 0.985 mg/mL; **2**, 1.000 mg/mL; **3**, 0.990 mg/mL; **4**, 1.075 mg/mL; **5**, 0.985 mg/mL; **6**, 0.990 mg/mL; **7**, 2.018 mg/mL; **8**, 5.060 mg/mL; **9**, 1.000 mg/mL; **10**, 1.235 mg/mL; **11**, 1.100 mg/mL; **12**, 1.170 mg/mL; **13**, 1.885 mg/mL; **14**, 0.976 mg/mL; **15**, 1.015 mg/mL; **16**, 1.025 mg/mL; **17**, 0.985 mg/mL; **18**, 1.004 mg/mL; **19**, 1.006 mg/mL; **20**, 1.180 mg/mL; and, **21**, 0.990 mg/mL. The mixed stock solution containing twenty-one reference compounds was further diluted with 72% *(v/v)* methanol to obtain a series of standard working solutions for the construction of calibration curves. All of the solutions were stored at 4 °C and then filtered through the 0.22 µm membranes before LC-MS analysis.

### 4.4. Preparation of Sample Solutions

The FF was divided into three parts as pericarp, stalk, and seed. Each part was ground into powder. Approximately 0.5 g of sample powder was accurately weighed and then soaked in 19 mL of 72% *(v/v)* methanol. After accurate weighing, ultra-sonication (500 W, 40 kHz) was performed for 60 min. Subsequently, the same solvent was added to compensate for the weight lost during extraction. The extract was centrifuged at 12,000 rpm (8050 g) for 10 min. The supernatant was subsequently collected, diluted tenfold, and then filtered through a 0.22 μm membrane prior to LC-MS analysis.

### 4.5. Chromatographic and Mass Spectrometric Conditions

Chromatographic analysis was conducted on a SHIMADZU UFLC XR system (Shimadzu Co., Kyoto, Japan), consisting of a LC-20AD binary pump, SIL-20A XR auto sampler, and a CTO-20AC column oven. Chromatographic separation was achieved on an XBridge^®^C18 column (4.6 mm × 100 mm, 3.5 μm, Waters, Ireland) and maintained at 30 °C with 0.1% *(v/v)* aqueous formic acid water solution (A)-acetonitrile (B) at a flow rate of 0.8 mL/min. The elution program was optimized as follows: 2% B at 0–0.01 min.; 2–23% B at 0.01–6.5 min.; 23–24% B at 6.5–10.0 min.; 24–45% B at 10.0–14.0 min.; 45–55% B at 14.0–16.0 min.; 55–2% B at 16.0–17.0 min. The column was equilibrated for additional 3 min. in 2% B before the next injection. The injection volume was 2 μL for each sample.

The mass spectrometric detection was performed on an API5500 triple quadrupole/linear ion trap mass spectrometer (AB Sciex, Framingham, MA, USA), which was equipped with an electrospray ionization (ESI) source operating under both positive and negative ion modes. The operation parameters of the mass spectrometer were set, as follows: the ion source temperature (TEM), 550 °C; the spray voltage (IS), 4500 V in the positive mode and −4500 V in the negative mode; the flow rate of curtain gas (GUR), 40 L/min.; the flow rate of nebulization gas (GS1), 55 L/min; and, the flow rate of auxiliary gas (GS2), 55 L/min. Quantitative analysis were obtained by MRM. All MS data were acquired by Analyst 1.6.3 software.

### 4.6. Validation of the Method

Validation of the method was carried out according to the International Conference on Harmonisation (ICH) guidelines Q2 (R1) [34], in terms of linearity, sensitivity, intra- and inter-day precision, repeatability, stability, accuracy, and matrix effect. A series of diluted mixed standard working solutions were analyzed from low to high concentration. Subsequently, plotting the peak area (*Y*) versus the corresponding concentration (*X*) of each analyte developed the calibration curves. The regression equation, correlation coefficient, and linear range were calculated; the LOD and LOQ of each analyte were measured at signal-to-noise ratio (S/N) of about 3 and 10, respectively. Intra-day precision was performed by analyzing the same mixed standard solution six times within one day, while the inter-day precision was examined by analyzing the solution three times a day for three consecutive days. Six independent samples from the same batch were parallel processed and analyzed to verify the repeatability. Stability was further evaluated on the same sample solution at 0, 2, 4, 8, 12, and 24 h after preparation, respectively. The variations of precision, repeatability, and stability were presented with RSD values of the peak area. For accuracy measurement, a recovery test was conducted by adding certain amounts of each reference solution (approximately equivalent to 80%, 100%, 120% of the known amounts) to the sample powder (0.25 g), which were repeated three times for each level. The average recovery was calculated, as follows: recovery (%) = (found amount − original amount)/spiked amount × 100%. Furthermore, the standard addition calibration curves were constructed by analyzing the extracts that were spiked with appropriate amounts of references. The slope ratio of standard addition calibration curve to solvent calibration curve was calculated to study the matrix effect [35].

### 4.7. Data Analysis

To get a good overview of the sample classification from different segments of FF, dendrogram of HCA was created according to the contents of twenty-one analytes (SPSS 22.0 for Windows, Chicago, IL, USA). The histograms and boxplots were charted by Origin 9.0 (OriginLab, Northampton, MA, USA). All of the experimental data were statistically compared by one-way ANOVA, and the significance threshold was set at α = 0.005.

## 5. Conclusions

In this study, an approach using UFLC-QTRAP-MS/MS combined with multivariate statistical analysis was established to map the distribution of bioactive constituents in three segments of FF. The three segments showed certain similarity in composition, but significant difference in abundance. Phenylethanoid glycosides covering the most part are their predominant compositions, followed by lignans, flavonoids, and phenolic acids. More specifically, phenylethanoid glycosides are found with comparatively higher abundance in the seed, lignans are detected in particular in the stalk, flavonoids are preferably distributed in the pericarp, but the abundance of phenolic acids in three segments is about the same. This research is helpful in further understanding the distribution patterns of bioactive constituents in FF, and also lays a good foundation for exploring the accumulation rule of metabolites in FF.

## Figures and Tables

**Table 1:** Optimized mass spectrometric parameters for MRM of twenty-one analytes.

No.	Analyte	Formula	*t_R_* (min)	MRM Parameters		
				MRM Transitions (*m/z*)	DP (V)	CE (eV)
1	Gallic acid	C_7_H_6_O_5_	2.99	169.0/125.0	−35	−15
2	Chlorogenic acid	C_16_H_18_O_9_	5.61	352.8/190.9	−31	−26
3	Caffeic acid	C_9_H_8_O_4_	6.40	179.0/134.6	−125	−20
4	Forsythoside B	C_34_H_44_O_19_	7.52	755.2/160.7	−60	−56
5	Forsythoside I	C_29_H_36_O_15_	7.62	623.2/160.9	−85	−50
6	Rutin	C_27_H_30_O_16_	7.64	609.2/300.1	−65	−56
7	*p*-Coumaric acid	C_9_H_8_O_3_	7.80	163.0/118.9	−56	−19
8	Forsythiaside A	C_29_H_36_O_15_	7.82	623.2/160.9	−85	−50
9	Galuteolin	C_21_H_20_O_11_	8.10	447.1/285.0	−50	−28
10	Ferulic acid	C_10_H_10_O_4_	8.31	193.0/133.9	−27	−24
11	(+)-Pinoresinol-4-*O*-*β*-D-glucoside	C_26_H_32_O_11_	8.67	519.2/357.1	−160	−22
12	Astragalin	C_21_H_20_O_11_	8.76	448.9/287.0	22	12
13	Quercetin	C_15_H_10_O_7_	8.87	447.0/301.0	−165	−30
14	Hesperidin	C_28_H_34_O_15_	9.37	609.3/301.0	−66	−35
15	Baicalin	C_21_H_18_O_11_	11.77	445.0/269.0	−25	−18
16	(+)-Phillyrin	C_27_H_34_O_11_	12.50	556.9/309.0	130	47
17	(−)-Arctiin	C_27_H_34_O_11_	12.87	556.9/395.1	130	47
18	Luteolin	C_15_H_10_O_6_	13.06	285.0/133.0	−50	−32
19	Kaempferol	C_15_H_10_O_6_	14.76	285.0/116.9	−120	−36
20	(+)-Pinoresinol	C_20_H_22_O_6_	15.38	357.1/121.0	−45	−28
21	(+)-Phillygenin	C_21_H_24_O_6_	15.80	371.2/356.0	−37	−12

**Table 2:** Regression equations, limits of detection (LODs) and limits of quantification (LOQs), precision, repeatability, stability, recovery test, and matrix effect of twenty-one analytes.

No.	Analyte	Regression Equation	*r*	Liner Range (ng/mL)	LOD (ng/mL)	LOQ (ng/mL)	Precision (RSD, %)		Repeatability (RSD, %) (*n* = 6)	Stability (RSD, %) (*n* = 6)	Recovery (%)		Matrix Effect
							Intra-day (*n* = 6)	Inter-day (*n* = 9)			Mean	RSD	
1	Gallic acid	*Y* = 6740*X* – 565,000	0.9992	123.200~1970	17.983	59.943	1.8	2.9	4.0	2.3	98.59	2.1	0.92
2	Chlorogenic acid	*Y* = 1780*X* + 11,100	0.9996	0.556~5560	0.111	0.371	3.6	2.8	3.8	2.8	100.9	2.4	1.06
3	Caffeic acid	*Y* = 5010*X* + 23,300	0.9993	0.799~1598	0.126	0.420	1.4	2.9	2.6	3.5	99.99	1.8	0.95
4	Forsythoside B	*Y* = 569*X* + 23,900	0.9992	2.690~107,600	0.359	1.196	2.1	3.7	2.2	3.7	100.3	1.2	0.93
5	Forsythoside I	*Y* = 0.813*X* + 749	0.9993	424~106,000	106.047	353.489	3.9	4.0	4.0	4.0	100.6	1.4	0.92
6	Rutin	*Y* = 434*X* + 56,000	0.9990	8.720~174,400	1.939	6.462	1.6	3.9	1.4	3.1	100.5	2.1	0.94
7	*p*-Coumaric acid	*Y* = 3710*X* + 3900	0.9991	0.060~238	0.007	0.024	2.0	3.8	4.0	3.8	99.90	1.7	0.91
8	Forsythiaside A	*Y* = 0.393*X* + 1880	0.9990	504~756,000	131.955	439.851	3.8	3.0	4.0	3.1	101.0	1.8	0.96
9	Galuteolin	*Y* = 5890*X* + 319	0.9992	0.003~3.220	0.001	0.002	2.7	3.0	3.9	3.8	100.8	2.8	1.02
10	Ferulic acid	*Y* = 482*X* + 125	0.9991	0.397~397	0.106	0.354	2.4	4.0	3.0	3.2	100.2	1.9	1.03
11	(+)-Pinoresinol-4-*O*-*β*-D-glucoside	*Y* = 645*X* + 396,000	0.9991	5.200~104,000	0.574	1.913	3.9	3.9	2.3	3.6	100.1	1.4	0.97
12	Astragalin	*Y* = 3970*X* + 1180	0.9993	0.180~72	0.040	0.132	1.2	3.7	3.8	3.8	100.8	1.9	1.02
13	Quercetin	*Y* = 8.23*X* + 1.39	0.9991	1.590~381	0.381	1.270	4.0	3.2	4.0	3.7	101.9	1.7	0.98
14	Hesperidin	*Y* = 7430*X* – 31,700	0.9996	4.333~976	0.090	0.300	4.0	3.5	3.6	3.9	100.5	2.3	0.95
15	Baicalin	*Y* = 5580*X* + 3590	0.9997	0.022~22.500	0.005	0.018	2.6	4.0	3.5	4.0	100.2	0.89	1.03
16	(+)-Phillyrin	*Y* = 1.75*X* + 272	0.9992	15.650~78,250	3.130	10.434	4.0	3.8	3.7	3.5	99.88	1.4	1.02
17	(–)-Arctiin	*Y* = 44.9*X* + 50.6	0.9990	0.171~34,200	0.034	0.114	3.6	3.5	3.9	4.0	101.6	1.5	0.97
18	Luteolin	*Y* = 5360*X* – 235,000	0.9992	43.850~4020	0.460	1.532	3.9	3.9	2.2	3.8	101.9	2.0	0.99
19	Kaempferol	*Y* = 194*X* + 211	0.9992	0.102~102	0.024	0.082	3.0	3.9	3.8	1.8	99.21	3.8	0.95
20	(+)-Pinoresinol	*Y* = 99.6*X* + 101,000	0.9993	0.259~13,000	0.063	0.211	2.8	3.5	3.9	4.0	100.3	2.3	1.02
21	(+)-Phillygenin	*Y* = 0.457*X* − 342	0.9990	792~39,600	233.710	771.250	3.9	3.2	3.9	3.6	100.7	2.6	0.97

**Table 3:** Sample information of Forsythiae Fructus.

Sample No.	Origin	Locality	Sample No.	Origin	Locality
S1	Shanxi, China	Pingshun	S8	Shanxi, China	Guxian
S2	Shanxi, China	Huguan	S9	Shanxi, China	Lingchuan
S3	Shanxi, China	Anze	S10	Shaanxi, China	Heyang
S4	Shanxi, China	Anze	S11	Henan, China	Linchuan
S5	Shanxi, China	Anze	S12	Henan, China	Luoyang
S6	Shanxi, China	Anze	S13	Henan, China	Neixiang
S7	Shanxi, China	Guxian	S14	Henan, China	Huixian

## Supplementary Materials

The following are available online, Figure S1: Effects of methanol concentration, liquid to material ratio and extraction time on extraction yields of forsythiaside A and phillyrin, Figure S2: Analysis of interaction effect among methanol concentration, liquid to material ratio and extraction time on the total extraction yield of forsythiaside A and phillyrin, Figure S3: Multiple-reaction monitoring (MRM) chromatograms of twenty-one analytes in reference solution, Figure S4: Chemical structures of twenty-one analytes, Table S1: Design and result of Box-Behnken, Table S2: Variance analysis and significant test for response surface, Table S3: Content of twenty-one analytes in the pericarp, Table S4: Content of twenty-one analytes in the stalk, Table S5: Content of twenty-one analytes in the seed.
