# Supplementary material for: Distribution Patterns for Bioactive Constituents in Pericarp, Stalk and Seed of Forsythiae Fructus
Source: Molecules. 2020 Jan 14;25(2):340. doi: 10.3390/molecules25020340 (PMC7024327; doi:10.3390/molecules25020340)
Supplement: Supplementary file 1 [file molecules-25-00340-s001.pdf]

Supplementary materials

Supplementary figures

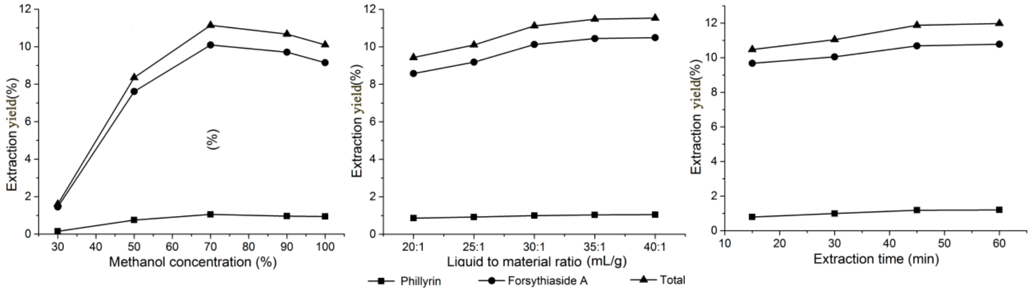

Figure S1. Effects of methanol concentration, liquid to material ratio and extraction time on extraction yields of forsythiaside A and phillyrin. ("extraction yield (%) = weight of analyte (g) / weight of dried sample (g) × 100%).

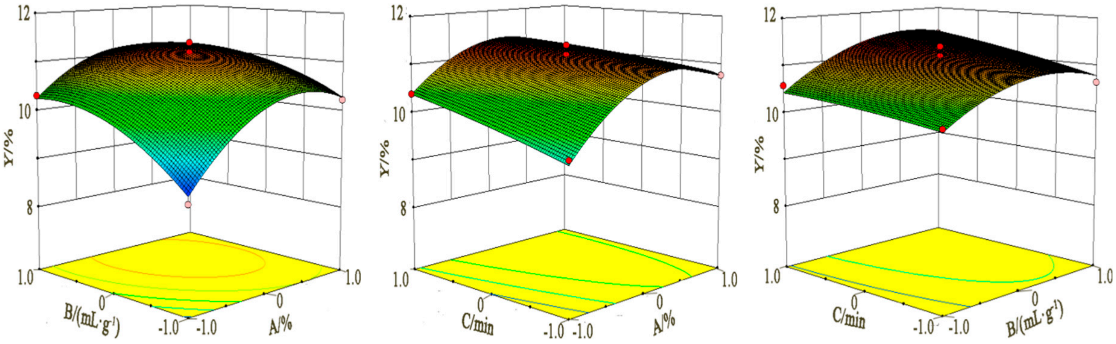

Figure S2. Analysis of interaction effect among methanol concentration, liquid to material ratio and extraction time on the total extraction yield of forsythiaside A and phillyrin.

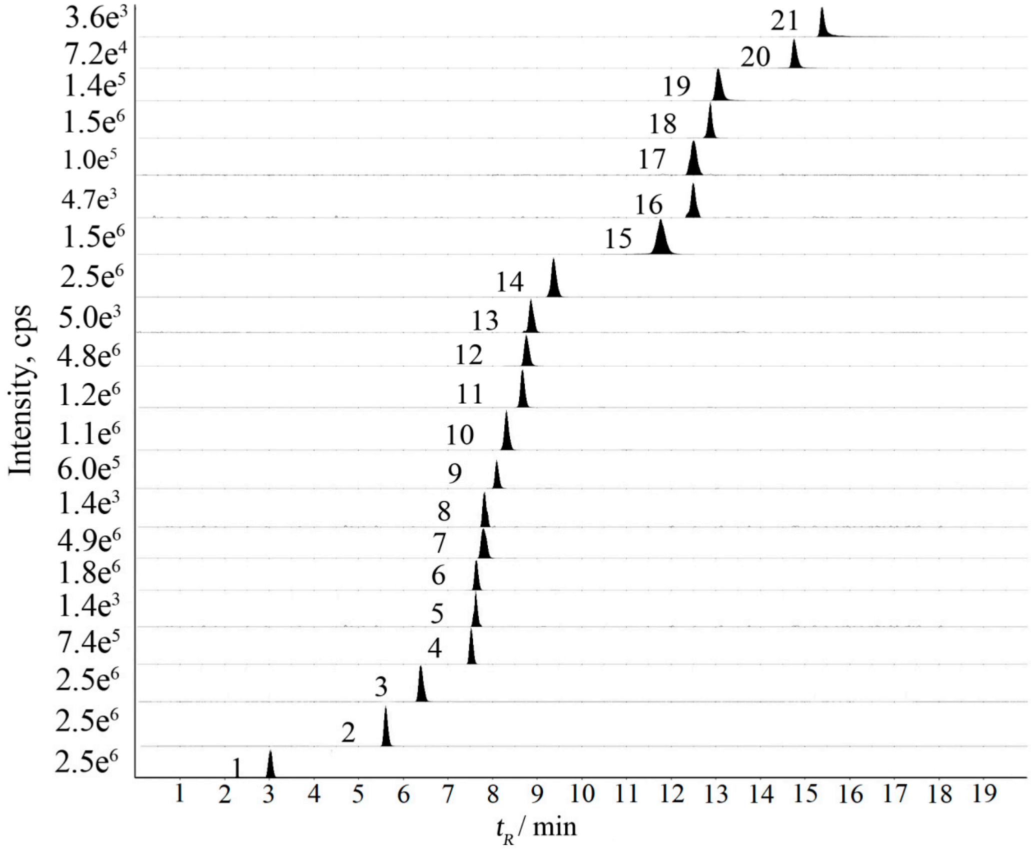

Figure S3. Multiple-reaction monitoring (MRM) chromatograms of twenty-one analytes in reference solution.

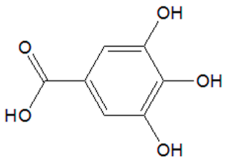

Gallic acid (1)

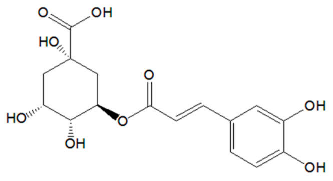

Chlorogenic acid (2)

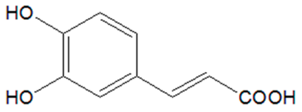

Caffeic acid (3)

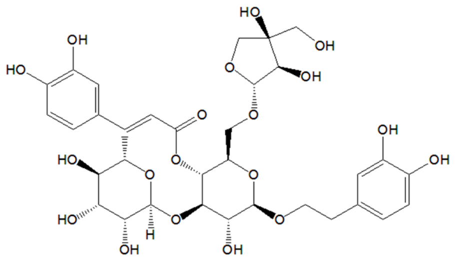

Forsythoside B (4)

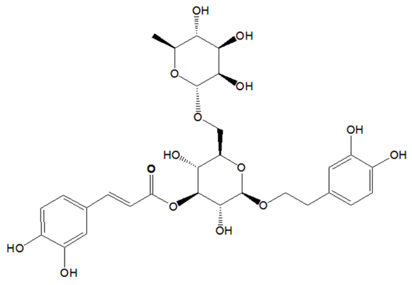

Forsythoside I (5)

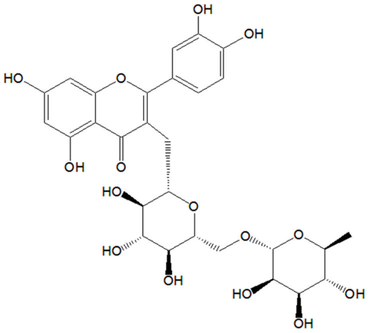

Rutin (6)

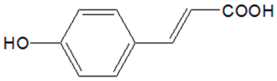

***p*-Coumaric acid (7)**

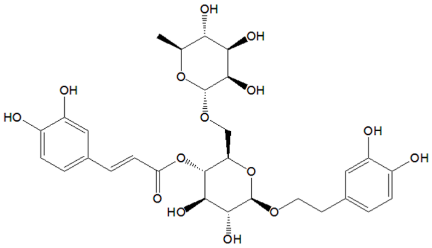

Forsythiaside A (8)

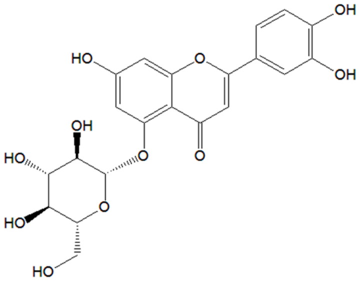

Galuteolin (9)

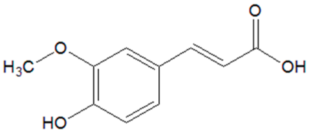

Ferulic acid (10)

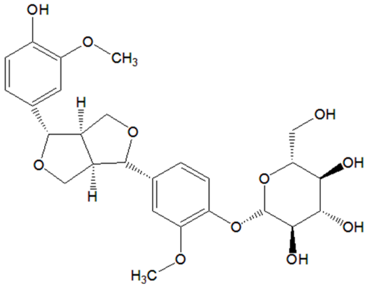

(+)-Pinoresinol-4-*O*-*β*-D-glucoside (11)

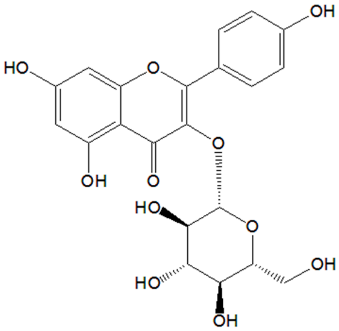

Astragalin (12)

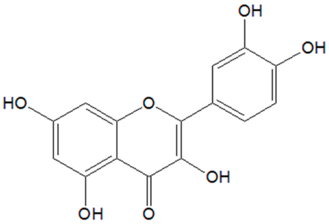

Quercetin (13)

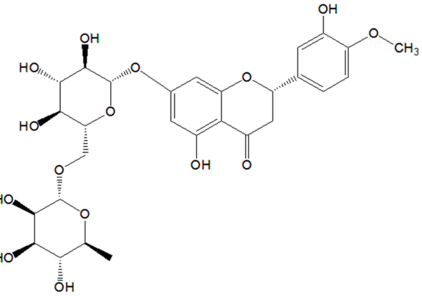

Hesperidin (14)

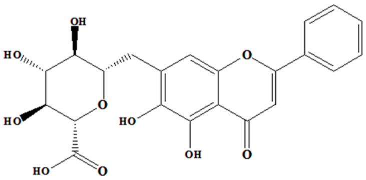

Baicalin (15)

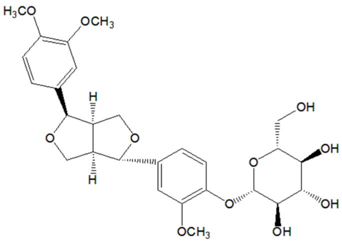

(+)-Phillyrin (16)

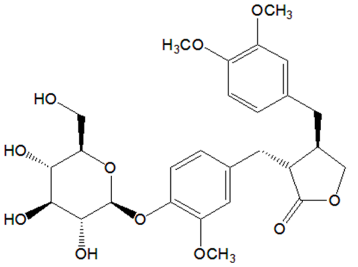

(−)-Arctiin (17)

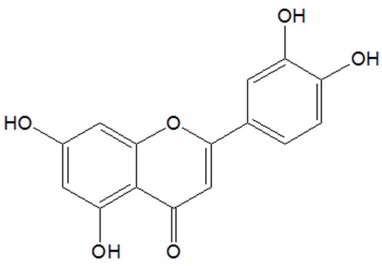

Luteolin (18)

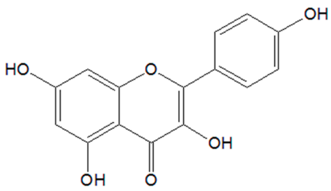

Kaempferol (19)

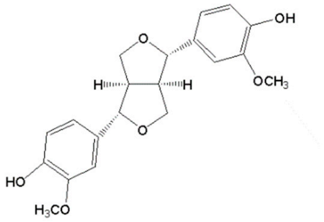

(+)-Pinoresinol (20)

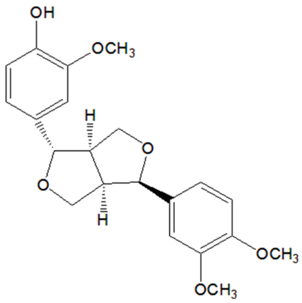

(+)-Phillygenin (21)

Figure S4. Chemical structures of twenty-one analytes.

Supplementary tables

Table S1. Design and result of Box-Behnken.

Run	X_1	X_2	X_3	Response value
	**Methanol concentration (%)**	**Liquid to material ratio (mL/g)**	**Extraction time (min)**	***Y* (%)**
1	70 (0)	40:1 (1)	30 (−1)	10.64
2	90 (1)	35:1 (0)	60 (1)	10.78
3	70 (0)	40:1 (1)	60 (1)	11.39
4	70 (0)	35:1 (0)	45 (0)	11.13
5	50 (−1)	30:1 (−1)	45 (0)	8.76
6	70 (0)	35:1 (0)	45 (0)	11.40
7	90 (1)	40:1 (1)	45 (0)	10.73
8	70 (0)	35:1 (0)	45 (0)	11.20
9	90 (1)	35:1 (0)	30 (−1)	10.77
10	50 (−1)	35:1 (0)	30 (−1)	9.64
11	90 (1)	30:1 (−1)	45 (0)	10.23
12	70 (0)	30:1 (−1)	60 (1)	10.58
13	50 (−1)	35:1 (0)	60 (1)	10.39
14	70 (0)	35:1 (0)	45 (0)	11.14
15	50 (−1)	40:1 (1)	45 (0)	10.31
16	70 (0)	30:1 (−1)	30 (−1)	10.21
17	70 (0)	35:1 (0)	45 (0)	11.07

Note: "*Y*" the total extraction yield of forsythiaside A and phillyrin. (*Y* (%) = weight of analyte (g) / weight of dried sample (g) × 100%).

Table S2. Variance analysis and significant test for response surface.

Source	Sum of squares	Degree of freedom	*F*-value	*p*-value	Source	Sum of squares	Degree of freedom	*F*-value	*p*-value
Model	7.04	9	30.42	<0.0001**	A^2	2.34	1	90.94	<0.0001**
A	1.45	1	56.52	0.0001**	B^2	0.80	1	31.02	0.0008**
B	1.35	1	52.62	0.0002**	C^2	9.60×10^{-3}	1	0.37	0.5605
C	0.44	1	17.18	0.0043**	Residual	0.18	7		
AB	0.28	1	10.72	0.0136*	Lack of fit	0.12	3	2.38	0.2108
AC	0.14	1	5.32	0.0544	Pure error	6.50×10^{-2}	4		
BC	3.60×10^{-2}	1	1.40	0.2747	Core total	7.22	16		

Note: $^{*}p \leq 0.05$; $^{**}p \leq 0.01$.

Table S3. Content of twenty-one analytes in the pericarp. (μg/g, *n* = 3).

Analyte	S1-P	S2-P	S3-P	S4-P	S5-P	S6-P	S7-P	S8-P	S9-P	S10-P	S11-P	S12-P	S13-P	S14-P
Gallic acid	34.86	33.98	34.56	33.57	33.33	34.14	33.16	33.28	32.78	34.68	33.56	34.57	34.89	32.97
Chlorogenic acid	17.59	36.51	30.92	36.04	30.91	55.75	27.29	26.04	31.76	56.34	41.62	10.39	48.25	25.40
Caffeic acid	41.58	59.71	88.44	208.97	93.73	146.28	94.52	109.04	294.56	69.23	72.12	73.26	73.75	57.44
Forsythoside B	85.64	497.91	167.59	276.39	287.01	300.89	374.57	453.13	366.42	269.87	240.54	245.63	56.20	248.03
Forsythoside I	3298.97	16018.69	9693.30	11141.38	12540.25	14106.83	13199.37	16626.68	7776.53	10774.15	12786.54	10999.05	3255.56	10829.55
Rutin	4780.17	11077.43	7537.69	9926.58	10624.50	11695.38	7740.50	8247.62	7571.18	9669.86	9321.50	5401.49	9150.01	9502.35
p-Coumaric acid	35.59	9.35	12.91	11.27	10.96	12.62	18.95	11.90	19.86	20.70	14.97	9.71	16.20	21.64
Forsythiaside A	58576.64	99768.77	74717.51	91435.22	90450.80	98840.83	91066.88	98801.27	101561.50	90329.77	100695.97	91223.71	67163.96	90208.55
Galuteolin	0.02	0.01	0.02	0.04	0.05	0.03	0.03	0.04	0.03	0.02	-	-	-	0.02
Ferulic acid	38.18	12.60	15.90	12.27	14.00	11.82	13.38	14.10	21.09	19.30	20.01	12.74	15.21	22.55
(+)-Pinoresinol-4-*O*-*β*-D-glucoside	3812.06	11969.23	9894.10	11012.80	9715.51	12091.97	10249.42	9905.98	10127.59	9487.63	9960.94	6006.85	4765.53	7490.52
Astragalin	1.14	1.88	1.47	1.36	2.13	1.76	1.67	1.75	1.71	1.81	1.74	0.66	1.43	2.44
Quercetin	13.66	22.67	8.38	10.46	22.96	5.48	5.38	11.39	13.78	13.28	9.03	6.95	41.19	10.06
Hesperidin	1.86	1.83	1.85	1.93	1.94	1.94	1.92	1.88	2.06	1.92	1.90	1.82	1.95	1.87
Baicalin	0.93	0.77	0.83	0.74	1.08	1.52	1.35	3.09	0.84	1.11	0.82	0.84	0.75	0.95
(+)-Phillyrin	1527.61	8849.10	6776.87	9315.92	7860.36	10852.36	8514.67	8436.28	7209.46	10689.51	6434.80	5430.31	4873.00	4613.20
(−)-Arctiin	11.01	32.51	14.46	24.35	33.99	43.03	17.17	23.62	10.48	44.51	9.98	11.83	4.97	8.72
Luteolin	16.75	16.72	16.73	16.66	16.68	18.49	17.47	17.04	17.05	17.06	16.75	16.77	16.80	16.80
Kaempferol	0.96	0.35	0.38	-	0.37	0.77	0.51	0.22	1.20	0.79	0.07	-	-	0.64
(+)-Pinoresinol	2069.94	1546.11	808.35	1715.84	1818.43	1928.64	1731.44	1179.62	2405.51	1972.49	2007.23	1624.00	1985.13	1970.25
(+)-Phillygenin	309.71	306.84	528.52	434.62	409.61	355.33	306.95	308.26	307.83	329.03	308.14	332.84	1416.08	308.32

Note: "-"not detected; "P" pericarp.

Table S4. Content of twenty-one analytes in the stalk. (μg/g, *n* = 3).

**Analyte**	**S1-ST**	**S2-ST**	**S3-ST**	**S4-ST**	**S5-ST**	**S6-ST**	**S7-ST**	**S8-ST**	**S9-ST**	**S10-ST**	**S11-ST**	**S12-ST**	**S13-ST**	**S14-ST**
Gallic acid	32.18	33.55	34.02	32.72	32.53	32.79	32.78	32.49	32.33	32.66	32.32	32.39	32.77	32.29
Chlorogenic acid	39.89	52.08	42.63	43.91	50.12	53.11	38.39	41.61	40.53	46.55	44.35	13.82	58.25	39.24
Caffeic acid	56.09	45.27	72.72	220.25	70.02	173.37	55.55	98.35	137.77	56.59	58.33	31.25	35.62	45.32
Forsythoside B	8063.27	9068.45	5021.20	4981.17	6544.96	5731.83	7127.06	7797.75	7862.96	6111.78	7190.94	7334.66	3022.10	7127.06
Forsythoside I	4299.73	9607.56	4693.19	4879.97	6423.43	7873.09	8293.58	10914.37	5631.57	6711.75	7169.39	3625.03	738.82	7125.54
Rutin	2533.41	4855.17	3458.57	5269.20	6435.13	4773.48	4029.54	4285.07	3706.45	5093.66	3319.28	1668.10	2559.67	4309.61
p-Coumaric acid	20.29	12.20	14.23	15.36	11.78	17.62	15.88	11.58	16.60	15.69	11.17	9.11	12.50	17.21
Forsythiaside A	64210.06	88026.77	66960.01	82898.27	79935.75	89037.16	81981.46	93520.61	84898.03	73936.47	86006.51	61649.20	46228.92	69319.86
Galuteolin	-	-	0.10	0.01	-	0.01	0.01	-	0.01	-	-	-	0.03	-
Ferulic acid	30.41	16.07	25.03	19.43	15.82	17.48	18.66	14.96	17.95	18.68	11.32	9.92	11.33	14.72
(+)-Pinoresinol-4-*O*-*β*-D-glucoside	19558.13	22099.80	20601.91	21425.89	21905.49	21320.96	21026.50	21447.32	21619.74	19986.44	21371.31	18984.29	13196.62	21203.18
Astragalin	1.21	0.97	0.74	0.95	1.19	0.96	1.12	1.27	0.81	1.24	0.82	0.35	1.26	1.13
Quercetin	14.02	6.68	44.68	9.90	9.30	3.26	4.92	9.17	4.03	23.82	8.52	4.03	9.54	13.60
Hesperidin	1.83	1.81	1.85	1.93	1.84	1.88	1.78	1.83	1.81	1.86	1.83	1.79	1.84	1.88
Baicalin	0.87	0.78	0.80	0.72	0.70	0.67	0.70	1.47	0.67	0.78	0.69	0.72	0.87	1.01
(+)-Phillyrin	4521.75	8215.72	5776.30	6123.39	7015.58	6865.05	6930.17	7150.08	6193.41	7567.19	5452.00	5742.10	2980.34	5323.95
(−)-Arctiin	300.80	491.39	415.55	392.72	442.78	413.26	405.65	449.82	348.19	403.51	287.94	440.77	224.65	286.36
Luteolin	16.70	16.74	17.00	16.73	16.77	16.75	16.70	16.75	16.73	16.87	16.67	16.74	16.76	16.71
Kaempferol	0.24	-	0.23	-	0.06	0.44	-	0.23	-	-	0.07	0.15	0.68	0.49
(+)-Pinoresinol	1933.95	1438.64	1151.06	1555.07	1559.51	1761.95	1220.40	1251.41	1403.74	1985.13	1448.64	1481.21	1048.99	583.50
(+)-Phillygenin	482.18	405.86	2119.89	844.80	747.91	839.49	901.00	384.16	333.04	934.35	355.85	881.10	1772.43	600.11

Note: "-"not detected; "ST" stalk.

Table S5. Content of twenty-one analytes in the seed. (μg/g, *n* = 3).

Analyte	S1-S	S2-S	S3-S	S4-S	S5-S	S6-S	S7-S	S8-S	S9-S	S10-S	S11-S	S12-S	S13-S	S14-S
Gallic acid	31.99	32.19	32.25	32.18	32.25	32.21	32.16	32.35	32.15	32.75	31.99	32.41	32.50	32.18
Chlorogenic acid	21.09	27.10	15.90	18.07	13.86	24.12	12.56	14.72	12.78	17.64	19.20	18.98	26.46	21.77
Caffeic acid	73.70	83.96	171.10	219.53	77.92	204.70	102.06	155.84	137.68	89.27	87.01	115.04	159.06	99.95
Forsythoside B	119.49	135.00	102.87	95.49	153.09	92.30	157.55	167.53	70.80	132.33	200.50	473.56	81.56	352.30
Forsythoside I	7634.90	22276.76	21095.35	16510.01	14802.71	16957.45	18518.25	22016.58	6235.33	15685.03	16623.35	17504.77	17835.52	15320.23
Rutin	6817.42	4951.50	5167.33	5812.68	6118.69	7890.00	5865.17	5033.03	3240.54	6037.43	3936.41	5003.04	3856.81	8494.63
p-Coumaric acid	15.77	14.05	18.13	15.57	12.82	17.44	14.54	13.52	14.95	16.09	13.64	16.50	26.03	18.36
Forsythiaside A	85119.97	105531.54	99669.04	101561.50	95898.76	108497.99	93156.01	102507.16	86103.13	98761.74	103618.04	120014.25	103597.31	111401.08
Galuteolin	-	-	-	-	-	-	0.01	-	-	0.02	0.01	-	-	0.03
Ferulic acid	16.36	9.99	9.59	6.43	10.87	7.07	7.86	9.90	9.51	10.78	14.33	19.22	11.41	29.73
(+)-Pinoresinol-4-*O*-*β*-D-glucoside	1161.82	6661.05	4342.64	5488.82	3769.27	5957.51	5276.90	4522.49	2056.84	4728.26	2577.96	2270.57	4144.89	4188.65
Astragalin	1.07	2.14	1.69	1.55	1.79	1.42	1.62	1.78	1.06	3.97	1.62	0.70	0.97	2.21
Quercetin	3.45	9.91	8.57	10.69	3.89	5.67	2.02	15.43	5.66	15.04	2.56	5.57	6.26	9.82
Hesperidin	1.85	1.78	1.82	1.82	1.81	1.75	1.83	1.78	1.80	1.84	1.80	1.83	1.76	1.81
Baicalin	1.01	0.92	0.95	0.83	0.83	0.93	0.93	1.25	0.97	0.82	1.17	1.04	0.54	0.38
(+)-Phillyrin	678.54	3654.81	2328.58	3954.92	2961.00	3504.88	3412.49	3173.19	1132.15	3372.47	1396.35	1649.85	1849.99	1451.24
(−)-Arctiin	0.56	13.37	2.44	3.20	5.66	7.75	2.74	5.47	1.53	7.07	3.96	3.42	4.15	27.27
Luteolin	16.74	16.81	16.77	16.67	16.71	16.73	16.75	16.67	16.68	16.74	16.72	16.69	16.76	16.78
Kaempferol	0.19	0.13	0.11	0.15	0.63	-	0.65	-	0.25	0.92	0.83	0.31	-	1.14
(+)-Pinoresinol	1577.94	1217.31	1186.07	690.01	1030.74	1282.95	617.58	956.67	1360.96	1118.10	683.21	1320.08	961.64	1004.22
(+)-Phillygenin	404.54	307.14	357.82	308.66	309.59	309.65	309.16	307.77	307.83	356.54	307.20	331.61	359.53	308.32

Note: "-"not detected; "S" seed.
